# High Light Intensity Triggered Abscisic Acid Biosynthesis Mediates Anthocyanin Accumulation in Young Leaves of Tea Plant (*Camellia sinensis*)

**DOI:** 10.3390/antiox12020392

**Published:** 2023-02-06

**Authors:** Chenxi Gao, Yue Sun, Jing Li, Zhe Zhou, Xuming Deng, Zhihui Wang, Shaoling Wu, Lin Lin, Yan Huang, Wen Zeng, Shiheng Lyu, Jianjun Chen, Shixian Cao, Shuntian Yu, Zhidan Chen, Weijiang Sun, Zhihui Xue

**Affiliations:** 1 College of Horticulture, Fujian Agriculture and Forestry University, Fuzhou 350002, China; 2 Anxi College of Tea Science, Fujian Agriculture and Forestry University, Quanzhou 362400, China; 3 Mid-Florida Research and Education Center, Department of Environmental Horticulture, Institute of Food and Agricultural Sciences, University of Florida, Apopka, FL 32703, USA; 4 Wuyixing Tea Industry Co., Ltd., Nanping 353000, China

**Keywords:** ABA, antioxidant activity, *Camellia sinensis*, purple leaves, ROS, transcriptome analysis

## Abstract

There is increasing interest in the production and consumption of tea (*Camellia sinensis* L.) processed from purple–leaved cultivar due to their high anthocyanin content and health benefits. However, how and why seasonal changes affect anthocyanin accumulation in young tea leaves still remains obscured. In this study, anthocyanin and abscisic acid (ABA) contents in young leaves of Zifuxing 1 (ZFX1), a cultivar with new shoots turning to purple in Wuyi Mountain, a key tea production region in China, were monitored over four seasons. Young leaves produced in September were highly purplish, which was accompanied with higher anthocyanin and ABA contents. Among the environmental factors, the light intensity in particular was closely correlated with anthocyanin and ABA contents. A shade experiment also indicated that anthocyanin content significantly decreased after 168 h growth under 75% shade, but ABA treatment under the shade conditions sustained anthocyanin content. To confirm the involvement of ABA in the modulation of anthocyanin accumulation, anthocyanin, carotenoids, chlorophyll, ABA, jasmonic acid (JA), and salicylic acid (SA) in the young leaves of four cultivars, including ZFX1, Zijuan (ZJ), wherein leaves are completely purple, Rougui (RG) and Fudingdabaicha (FDDB) wherein leaves are green, were analyzed, and antioxidant activities of the leaf extracts were tested. Results showed that ABA, not other tested hormones, was significantly correlated with anthocyanin accumulation in the purple–leaved cultivars. Cultivars with higher anthocyanin contents exhibited higher antioxidant activities. Subsequently, ZFX1 plants were grown under full sun and treated with ABA and fluridone (Flu), an ABA inhibitor. ABA treatment elevated anthocyanin level but decreased chlorophyll contents. The reverse was true to those treated with Flu. To pursue a better understanding of ABA involvement in anthocyanin accumulation, RNA–Seq was used to analyze transcript differences among ABA– or Flu–treated and untreated ZFX1 plants. Results indicated that the differentially expressed genes in ABA or Flu treatment were mainly ABA signal sensing and metabolism–related genes, anthocyanin accumulation-related genes, light–responsive genes, and key regulatory MYB transcription factors. Taking all the results into account, a model for anthocyanin accumulation in ZFX1 cultivar was proposed: high light intensity caused reactive oxygen stress, which triggered the biosynthesis of ABA; ABA interactions with transcription factors, such as MYB-enhanced anthocyanin biosynthesis limited chlorophyll and carotenoid accumulation; and transport of anthocyanin to vacuoles resulting in the young leaves of ZFX1 with purplish coloration. Further research is warranted to test this model.

## 1. Introduction

Plants as sessile organisms have evolved various mechanisms to counteract environmental stressors. Environmental stressors include extreme light intensities or temperatures, drought, and salinity. These stressful factors trigger plants to produce reactive oxygen species (ROS), which can lead to the damage of cell machinery [1]. To minimize ROS damages, the first line of plant defense is the production of antioxidants. Antioxidants are able to quench ROS and reduce the risk of oxidative damage. Plant antioxidants are a range of compounds including polyphenols and terpenoid. Polyphenols are a large group of plant secondary metabolites, of which anthocyanins are important pigments exhibiting red, purple, or blue color. Anthocyanins are biosynthesized through an extension of the general flavonoid pathway. Structural genes encoding different enzymes catalyze each reaction step, and regulatory genes encoding transcription factors modulate the expression of the structural genes [2]. Genetic makeup and developmental stages of plants as well as environmental factors regulate anthocyanin metabolism, and their content relies on the balance between biosynthesis and degradation. Anthocyanins are generally accumulated in the vacuole of cells in both vegetative and reproductive organs. Thus, anthocyanin biosynthesis has been considered a protective mechanism against environmental stressors.

Anthocyanins also have nutraceutical and pharmaceutical value. Clinic studies have shown that consuming food and beverages rich in anthocyanins alleviate cardiovascular and neurodegenerative diseases [3]. Tea (*Camellia sinensis* L.) is one of the most popular non-alcoholic beverages in the world. It has been reported that more than three billion people consume tea due to its variety of flavors and high levels of polyphenols, mainly anthocyanins. To increase anthocyanin contents, purple-leaved tea cultivars have been developed. Purple–leaved tea has a unique color and high content of bioactive anthocyanin [4]. Rashid et al. reported that tea anthocyanins could cross the blood–brain barrier and enhance the antioxidant capacity in mice brain [5]. In addition, kombucha made by purple–leaved tea (Zijuan) presented higher antioxidant activity [6]. Several newly developed cultivars including Zixin (ZX) [4], Zijuan [7], Ziyan [8], Jinmingzao [9], and Zifuxing1 (ZFX1) [10] have high content of anthocyanins. Some cultivars have completely purplish leaves, such as Ziyan and ZX, whereas other cultivars such as ZFX1 are green–leaved plants, but new emerging young leaves can turn to purplish at certain times of a year.

Molecular mechanisms underlying the change in tea leaf color have been studied. For example, purple leaves in Zijuan were attributed to the activation of R2R3–MYB transcriptional factor (TF) anthocyanin1 (*CsAN1*) that upregulated bHLH TF *CsGL3* and anthocyanin late biosynthetic genes (LBGs). The interaction among *CsAN1*, bHLHTFs, and WD-repeat protein *CdTTG1* regulated anthocyanin accumulation [7]. In purple–leaved ‘Ziyan’, UV–A and UV–B can induce anthocyanin accumulation by upregulation of both structural and regulatory genes involved in anthocyanin biosynthesis [11]. However, why newly emerging young leaves became purplish at the certain time of a year, and what the key environmental factors were that promoted the color changes, were largely obscured.

High light has been reported to trigger anthocyanin biosynthesis [12]. When light intensity exceeds the plant’s ability to use or dissipate the energy, excess light produces ROS that can impair a photosynthetic apparatus, resulting in reduced photosynthetic activities. In the meantime, ROS also trigger plants to synthesize anthocyanins to reduce the severity of photooxidative damage [13]. Anthocyanins have been shown to contribute substantially to H_2_O_2_ scavenging as compared to other phenols [14]. Additionally, anthocyanin accumulation in the fruit skin is correlated with plant hormones [15,16]. Notably, ABA has been confirmed to be a major regulator of anthocyanin synthesis in non-climacteric fruits (e.g., strawberry, grapes, and bilberry) [17,18,19]. Studies on the dynamic transcriptional landscape under a high light without heat stress showed that *Arabidopsis* seedling activated ABA synthesis after 6 h exposed to the high light followed by the induction of anthocyanin accumulation, and during which ABA receptor were downregulated after high light treatment [20]. In tea plant production, light has been shown to increase anthocyanin content in the dark-purple tea cultivar ‘Ziyan’ (*Camellia sinensis*) [11]. ABA is also associated with anthocyanin accumulation during tea plant growth and development. When Chen et al. compared the expression of MYBs between purple and green-leaved teas, the authors found that the expression of *CsMYB17* was prominent in the purple leaves, and such an expression level was also evident in leaves treated with exogenous application of 100 μM ABA [21].

To better understand environmental factors, plant hormones, and molecular mechanisms that influence leaf color changes, this study monitored key environmental factors in four seasons and identified high light intensity was a key factor affecting leaf color change in cultivar ZFX1. Further studies under shade and treatment with ABA and ABA-inhibitor Flu showed that anthocyanin accumulation in young leaves was a response of plants to high light stress, and anthocyanin biosynthesis was associated with ABA content. We also compared transcriptional expression profiles of leaves treated with ABA, Flu or water using RNA–Seq. Based on available data, a model was proposed to illustrate the interaction among light, ABA, and anthocyanin biosynthesis in the alleviation of high light stress in ZFX1 plants.

## 2. Materials and Methods

### 2.1. Monitoring Key Environmental Factors and Anthocyanin and ABA Contents in Young Leaves of ZFX 1 Cultivar

ZFX1, a cultivar producing purplish young leaves (first four leaves from shoot tips) depending on seasonal changes, was used in this study. ‘ZFX1’ tea plants were grown in a tea plantation in Wuyi Mountain, Fujian Province, China (117°95′ E 27°71′ N, 150–200 m above sea level), a key tea production region in China. Light intensity, precipitation, and temperature were monitored over a four-season period. Monthly averages of these parameters were calculated. Young leaves, i.e., four leaves from shoot tips were collected from randomly selected three plants in April, June, September, and November. The collected leaves were immediately frozen in liquid nitrogen and stored at −80 °C for analysis of anthocyanin and ABA, respectively. Pearson correlation coefficient was used to determine relationships among the environmental parameters and anthocyanin and ABA contents.

### 2.2. Effects of Shade Treatment with and without ABA on Pigment Contents in Young Leaves of ZFX1 and the Extracts’ Antioxidant Activities

In order to be certain that high light intensity was a key factor influencing anthocyanin accumulation in young leaves, ZFX1 plants were grown under full sun or under 75% shade in spring. Those grown under the shade were sprayed with or without a 100 μM ABA solution once. Young leaves were collected from randomly selected three plants per treatment before ABA application and 8, 72, and 168 h after applications, respectively. Leaf anthocyanin, total chlorophyll, and carotenoid contents were analyzed. Antioxidant activities of the extracts were also determined by 1,1–diphenyl–2–picrylhydrazyl (DPPH), 2,2′–azinobis–3–ethylbenzthiazoline–6–sulfonic acid (ABTS), and ferric ion reducing antioxidant power (FRAP).

### 2.3. Analysis of Pigments and Some Hormones in Young Leaves of Four Tea Cultivars and the Extracts’ Antioxidant Activities

To explore if the relationship between anthocyanin and ABA occurred in other cultivars with different degrees of purplish leaves, as well as if anthocyanin accumulation was also associated with jasmonic acid (JA) and salicylic acid (SA), four cultivars including ZFX1, Zhijuan (ZJ), in which leaves are completely purple, Rougui (RG) and Fudingdabaicha (FDDB), whose leaves are green, were investigated. These cultivars were grown in the same tea plantation mentioned above. Young leaves were collected from three randomly selected plants per cultivar in September. Leaf chlorophyll, carotenoid, ABA, JA, and SA contents were analyzed. Antioxidant activities of their extracts were determined. Relationships of the pigments with the hormones, anthocyanin, and antioxidant activities were analyzed by the Pearson correlation coefficient.

### 2.4. Analysis of Pigment Contents in Young Leaves of ZFX1 Treated with ABA and ABA- Inhibitor and the Extracts’ Antioxidant Activities

Plants of ‘ZFX1’ were pruned to the same extent. When new shoots appeared, the plants were sprayed with deionized water, 100 μM ABA and 50 μM Flu in April, respectively. The use of ABA and Flu concentrations was based on the previous studies [21,22]. There were 16 plants per treatment, which were randomly divided into four group, three each. Young leaves were collected from three plants of a randomly selected group before the treatment and 8 h, 72 h, and 168 h thereafter from the other groups, respectively. Leaf samples per treatment were divided into two portions. One portion was used for analysis of anthocyanin, ABA, total chlorophyll, and carotenoid contents. Antioxidant activities of the extracts were also determined. The remaining portion per treatment was used for extraction of RNA.

### 2.5. Analyses of Pigments, Hormones, and Antioxidant Activities

Anthocyanin content was determined according to the method described by Neff and Chory [23] with minor modifications. Tea samples were freeze–dried in a lyophilizer (Alphal–4 LD PLUS; Marin Christ, Germany) and freeze–drying time was 30 h. Ten mLof methanol in a 1% (*v*/*v*) HCl solution was added to 0.5 g ground, lyophilized tea leaves, respectively. After sonicated for 30 min, the extraction was centrifuged for 10 min at 10,000 rpm, and the aqueous phase was subjected to spectrophotometric quantification at 530 and 657 nm. The relative unit was calculated by the formula OD = A530 − 0.25 × A657. The relative anthocyanin content was expressed as mg/dry weight (DW).

Concentrations of chlorophyll and carotenoids were measured according to methods described by Lichtenthaler and Buschmann [24]. An aliquot of the ground sample (0.20 g) was mixed with 15 mL of 95% ethanol and incubated in darkness for 30 min. The chlorophyll extract was filtered and analyzed with a UV-5800pc ultraviolet spectrophotometer (Metash, Shanghai, China). The ultraviolet absorption wavelengths of chlorophyll a and b and total carotenoids were recorded as follows: absorption peaks of 665 nm for chlorophyll a, 649 nm for chlorophyll b, and 470 nm for total carotenoids. Concentrations of chlorophyll a (Chl a mg/g fresh weight), chlorophyll b (Chl b), total chlorophyll (total Chl), and total carotenoid (Car) were calculated.

The extraction of JA, SA, and ABA from tea leaves was based on a previously described method [25] with modifications. The extracting solutions were filtered through a 0.22 μm Millipore filter before analysis on a Waters UPLC–PDA–QDa system. Separation was achieved using Waters’ C_18_ column (100 mm × 2.1 mm, 1.7 μm) in 35 °C. The mobile phases A and B were 0.1% formic acid and methanol, respectively. The flow rate of the mobile phase was 0.25 mL/min and gradient elution procedures were set as follows: 40% B (8 min), 100% B (2 min), and 40% B (2 min).

In vitro antioxidant activities were measured by three methods [26,27]: 1,1–diphenyl–2–picrylhydrazyl (DPPH), 2,2′–azinobis–3–ethylbenzthiazoline–6–sulfonic acid (ABTS), and ferric ion reducing antioxidant power (FRAP), respectively. Ground leaf powder in 0.1 g was extracted by 5 mL distilled water to collect the supernatant after centrifugation at 8000× *g* for 10 min. The absorbance was determined by Multiskan FC series microplate reader (Thermo Scientific, Waltham, MA, USA) at wavelengths of 517 nm, 405 nm, and 593 nm for obtaining antioxidant activities analyzed by DPPH, ABTS, and FRAP, respectively.

### 2.6. Data Analysis of Aforementioned Experiments

All experiments mentioned above were arranged as completely randomized design with three replications. All data were subjected to analysis of variance (ANOVA) using SPSS (Version 21, SPSS Inc., Chicago, IL, USA). If significance occurred, mean differences were separated using Duncan test at *p* < 0.05 level. Data were reported as the mean ± standard deviation.

### 2.7. Transcriptomic Analysis Leaves Treated with ABA, Flu or Water

Leaf samples collected from ‘ZFX1’ plants sprayed with deionized water, ABA, and Flu after 8, 72, and 168 h of treatment were used for total RNA extraction. As there were three biological replications, total RNA was extracted from a total of 27 samples using the RNAprep Pure Plant Kit (Tiangen, Beijing, China), respectively. RNA concentration and purity were measured using Nano Drop 2000 (Thermo Fisher Scientific, Wilmington, DE, USA). In brief, raw data were obtained from the Illumina platform. The reads containing adapter and low–quality reads from raw data were removed to obtain clean reads. All clean reads were mapped to the reference transcripts of *C. sinensis* (http://tpia.teaplant.org/index.html, accessed on 1 December 2021) by applying TopHat2 [28]. Gene expression levels were calculated based on the number of reads mapped to the reference sequence to receive unigenes by using HISAT2 and String Tie software. DEGs were screened according to parameters fold-change ≥ 2 and *p*–value ≤ 0.05 by applying DEseq2 [29]. k–Means cluster analysis was conducted using an R package [30]. WGCNA (v1.66) was used to construct unsigned co–expression networks based on the gene expression matrix (Fragments Per Kilobase of exon model per Million mapped fragments, FPKM ≥ 1 in at least one of the samples). The parameters were as follows: the soft threshold power was 3; the min module size was 30; the cut height was 0.25. The co–expression network was visualized with Cytoscape (v3.8.2).

### 2.8. Quantitative RT-PCR Analysis of Candidate Genes Involved in Anthocyanin Biosynthesis

Based on transcriptome results, eight genes were selected for qRT–PCR analysis to examine the different expression profiles. qRT–PCR detection system of the samples was based on the previously described method [31]. The relative expression of each gene was calculated after normalization with *CsGAPDH* (XM_028237220.1) gene, which is publicly available in the National Center for Biotechnology Information (NCBI). The samples from the three independent biological replicates were used for the analyses. The expression levels of candidate genes were determined using the 2^−ΔΔCT^ method [32]. The specific primers used for RT–qPCR are listed in Table S1.

## 3. Results

### 3.1. Environmental Factors Related to Anthocyanin and ABA Contents in Young Leaves of ZFX1

**Figure 1:**
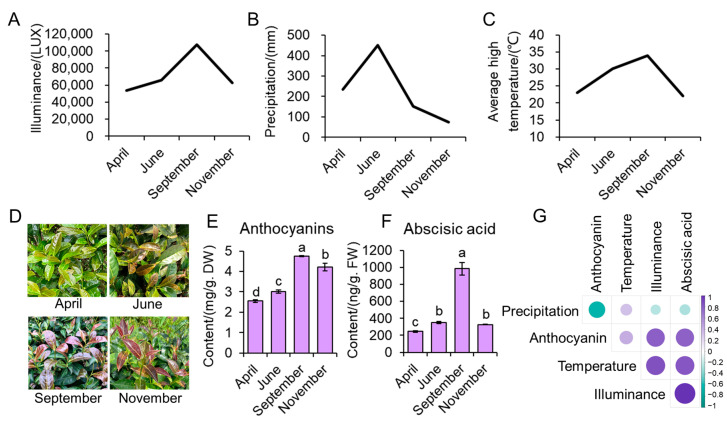
Changes in environmental factors in Wuyi Mountain tea plantation and young leaf color and anthocyanin and ABA content of ZFX1 plants over four seasons in 2020, which include mean illumination (**A**), precipitation (**B**), and temperature (**C**) as well as leaf color changes (**D**), anthocyanin (**E**) and ABA content (**F**), and correlation analysis of the factors with anthocyanin and ABA contents (**G**). The data in the graphs are mean values ± SD of three experimental replicates. Error bars are standard deviation of three samples (*n* = 3). Different letters above bars indicate significant differences among treatments analyzed by Duncan’s test at *p* < 0.05 level.

Key environmental factors recorded in Wuyi Mountain tea plantation in 2020 are shown in Figure 1. Solar radiation levels increased from 53,993 Lux (about 999 µmol m^−2^ s^−1^) in April to 107,332 Lux (about 1986 µmol m^−2^ s^−1^) in September and decreased in November (Figure 1A). The difference based on the PPFD was almost two-fold between April and September. June had the highest precipitation with a monthly mean of 451 mm, while November had the lowest average monthly precipitation of 73 mm (Figure 1B). Month mean temperature followed the radiation: increased from April to September, and decreased in November (Figure 1C). The highest average temperature was 34 °C in September compared to 23 °C in April. Young leaves of ZFX1 were green, became light purple in June, deep purple in September, and reduced purple color in November (Figure 1D). Such changes in color were accompanied with the changes in anthocyanin and ABA contents (Figure 1E,F). Anthocyanin contents were 2.6 mg/g DW in April, increased to 3.0 mg/g DW, jumped to 4.8 mg/g DW in September, then reduced to 4.2 mg/g DW. ABA contents increased from April to June, peaked to 1000 mg/g DW in September, and drastically decreased in November. Correlation coefficient analysis showed that ABA was highly correlated with the levels of illuminance or radiation, temperature, and anthocyanin. The anthocyanin content was significantly correlated with ABA and radiation, not significantly with temperature (Figure 1G).

### 3.2. Anthocyanin Contents Affected by Shade Treatment and ABA Application

**Figure 2:**
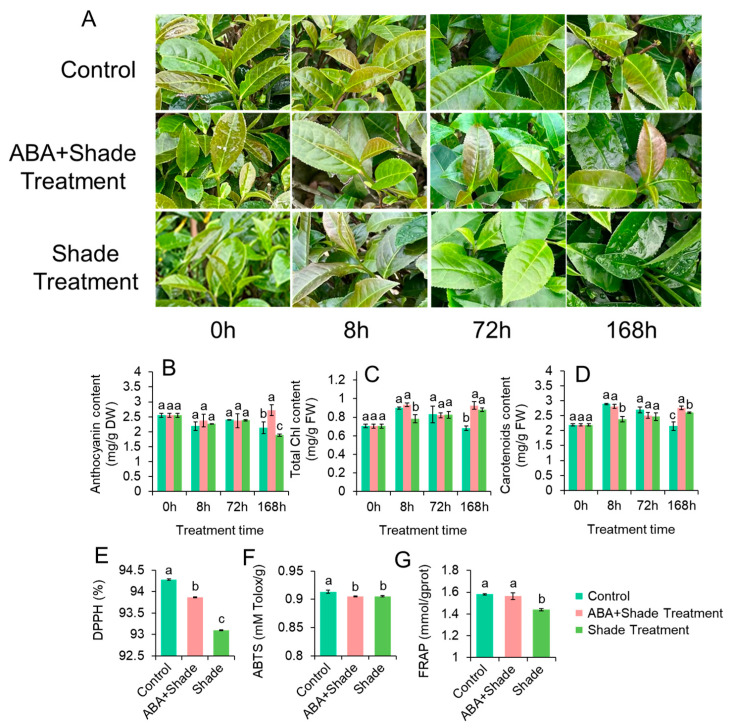
Effect of shade with or with ABA treatment on leaf color and pigment biosynthesis. Changes in leaf color of ZFX1 plants grown under full sun and 75% shade treated with or without ABA (**A**), anthocyanin (**B**), total chlorophyll (**C**), and carotenoid (**D**) contents of young leaves as well as antioxidant activities of leaf extracts analyzed by DPPH (**E**), ABTS (**F**), and FRAP (**G**). The data in the graphs are mean values ± SD of three experimental replicates. Error bars are standard deviation of three samples (*n* = 3). Different letters above bars indicate significant differences among treatments analyzed by Duncan’s test at *p* < 0.05 level.

To further test the role of high–light triggered anthocyanin accumulation and leaf color changes, a shade experiment was conducted in spring with ZFX1 grown under 75% shade (a PPFD of 749 µmol m^−2^ s^−1^). The leaf color of plants treated with or without shade remained green, but shaded plants treated with ABA produced purplish colored leaves 168 h after the treatment (Figure 2A). The appearance of purple leaves was accompanied with an increased level of anthocyanin in 168 h (Figure 2B), but the anthocyanin contents in the other two treatments were significantly lower. The changes in coloration also affected total chlorophyll contents (Figure 2C) in 168 h and carotenoid contents (Figure 2D) 8 and 168 h after the treatments. We also analyzed the antioxidant abilities of leaves collected 168 h after treatments. The shading treatment reduced antioxidant activities of tea leaves analyzed by DPPH (Figure 2E) and ABTS (Figure 2), but antioxidant activities of leaves treated with shade and ABA were significantly higher than shade treatment only (Figure 2E) or comparable with the control (Figure 2G). These results indicated that even under reduced light level, ABA plays an important role in modulate anthocyanin biosynthesis.

### 3.3. Anthocyanin and ABA Contents Were Substantially Higher in Purple Leaved Tea Cultivars

**Figure 3:**
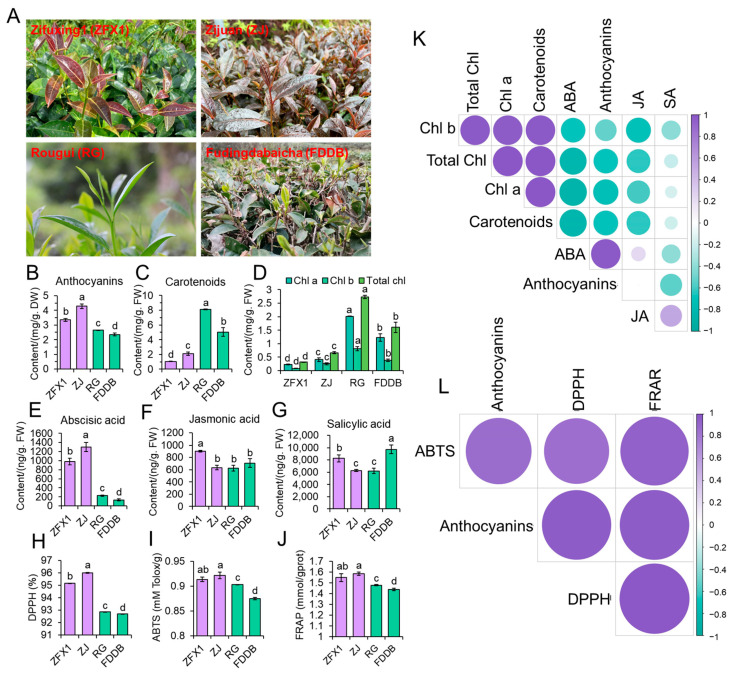
The close relationship between anthocyanin and ABA occurred in another purple leaved tea cultivar. Four tea cultivars ZFX1, ZJ, RG, and FDDB with purple, deep purple, green, and deep green leaves, respectively (**A**). Anthocyanin (**B**), carotenoid (**C**), chlorophyll (**D**), ABA (**E**), JA (**F**), and SA (**G**) contents in young leaves of four cultivars. Antioxidant activities of the young leaf extracts of the cultivars tested with DPPH (**H**), ABTS (**I**), and FDDB (**J**), respectively. Correlation analyses of the relationships among hormone and anthocyanin (**K**) as well as among antioxidant activities and anthocyanin (**L**). The data in the graphs are mean values ± SD of three experimental replicates. Error bars are standard deviation of three samples (*n* = 3). Different letters above bars indicate significant differences among treatments analyzed by Duncan’s test at *p* < 0.05 level.

To determine if the close relationship between anthocyanin and ABA occurred in other tea cultivars, four cultivars with different degrees of purpleness were compared (Figure 3A). The highest anthocyanin content (4.3 ± 0.15 mg/g DW) was observed in ZJ, followed by ZFX1 (3.5 ± 0.1 mg/g DW), as they have purple leaves. In contrast, anthocyanin contents in RG and FDDB were 2.7 ± 0.002 mg/g DW and 2.4 ± 0.091 mg/g DW, respectively (Figure 3B), because they are green–leaved cultivars. On the other hand, total carotenoids (Figure 3C) and chlorophyll a, b, and total chlorophyll (Figure 3D) in purple-leaved cultivars were significantly lower than green–leaved cultivars. ABA content in ZJ was the highest (1301 ± 104.86 ng/g FW), followed by ZFX1 (983 ± 73.70 ng/g FW), which was 4 ~ 10 times higher than that of green leaf cultivars RG (228 ± 14.06 ng/g FW) and FDDB (130 ± 23.00 ng/g FW) (Figure 3E). However, JA was the highest in ZFX1 (Figure 3F) and SA was the highest in FDDB (Figure 3G). Additionally, leaves with higher anthocyanin contents exhibited higher antioxidant activities tested by DPPH (Figure 3H), ABTS (Figure 3I), and FRAP (Figure 3J), respectively. Correlation analysis showed that anthocyanin accumulation was significantly correlated with ABA (r = 0.97), not with JA and SA (Figure 3K), and also closely associated with antioxidant activities (Figure 3L).

### 3.4. ABA and Flu Affected Anthocyanin Contents and Leaf Morphology of ZFX1

**Figure 4:**
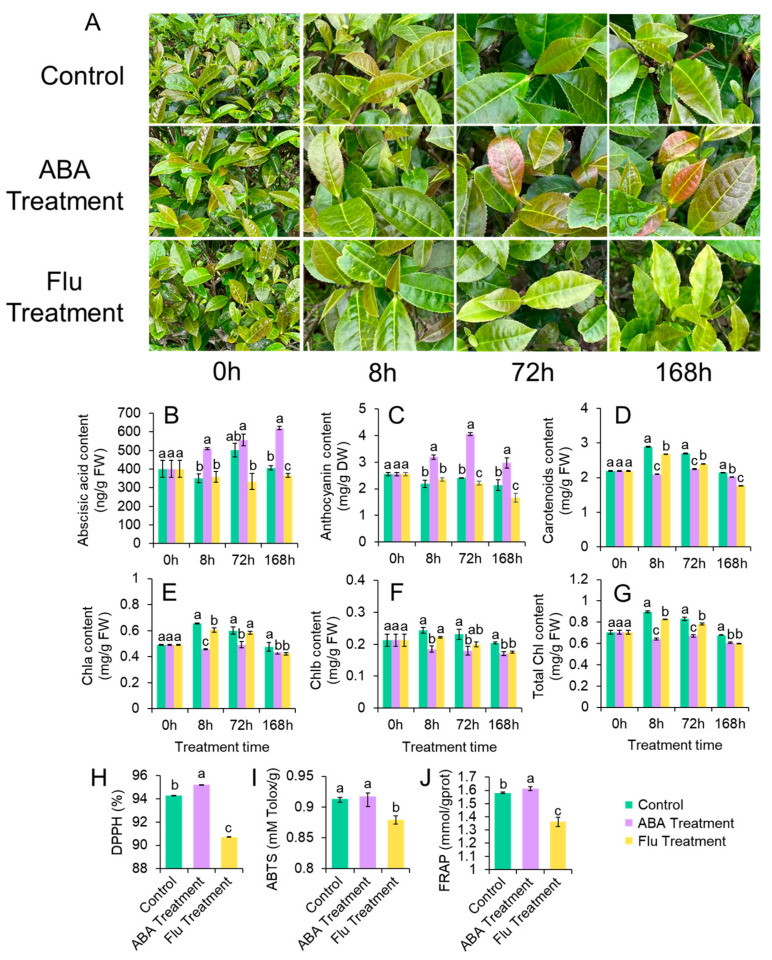
Changes in leaf color, ABA, and pigment contents in ZFX1 plants treated with ABA, Flu or water as well as antioxidant activities of leaf extracts harvested after 168 h of treatment. Leaf color changes in plants sprayed with water, ABA or Flu (**A**). ABA (**B**), anthocyanin (**C**), carotenoid (**D**), chlorophyll a (**E**), b (**F**), and total chlorophyll (**G**) contents in leaves treated with water, ABA or Flu. Antioxidant activities of leaves collected 168 h after treatments analyzed by DPPH (**H**), ABTS (**I**), and FPAP (**J**). The data are mean ± SD of three experimental replicates. Error bars show standard deviation (*n* = 3). Different letters above bars indicate significant differences based on Duncan’s test at *p* < 0.05) level.

To further investigate the relationship between leaf color and ABA, tea leaves of ZFX1 plants were treated with ABA, Flu or deionized water in spring. Compared to the control (water treatment), leaves of ABA treated plants became purplish after 8 h treatment, and the purple color intensified thereafter (Figure 4A). On the other hand, leaves treated with Flu stayed in green, and some leaves exhibited reduced greenness. Compared with the control plants, ABA content in young leaves increased from 11.0% at 8 h to 52.5% at 168 h after ABA treatment (Figure 4B), while the ABA content in Flu-treated leaves significantly decreased by 34.6% compared to the control. Anthocyanin content started to increase after 8 h of ABA treatment and reached the maximum at 72 h (4.1 ± 0.06 mg/g DW), which was 1.69–fold higher than the control (Figure 4C). On the contrary, anthocyanin levels in Flu-treated leaves began to decrease at 72 h and reached the lowest level (1.7 ± 0.16 mg/g) by 168 h. Carotenoid content decreased significantly from 8 to 72 h after ABA treatment and returned to the control level at 168 h (Figure 4D). ABA treatment decreased total chlorophyll, chlorophyll a, and b contents (Figure 4E–G). Flu treatment generally decreases carotenoid and chlorophyll contents. Leaf samples collected at 128 h were used for evaluating antioxidant activities. The antioxidant activities determined by DPPH, ABTS, and FRAP (Figure 4H–J) methods increased significantly after ABA treatment compared to that of the control. The antioxidant activities of leaves treated with Flu were significantly lower than those treated with water or ABA.

### 3.5. RNA–Seq and Functional Annotation

To elucidate the molecular mechanism behind the ABA–modulated anthocyanin biosynthesis, Illumina–based RNA–Seq was used to detect gene expression patterns among ZFX1 plants after 7, 72, and 168 h treatments with water, ABA, or Flu. A total of 27 samples generated 176.46 Gb clean data by RNA sequencing. More than 92.49% of clean data achieved a quality score of Q30 (Table S2). Based on gene expression, the correlation heat map analysis and principal component analysis (PCA) showed a good correlation among three biological replicates, and the samples in the same group were relatively consistent (Figure S1). In order to fully understand the function of genes obtained from transcriptome analysis, all unigenes were blasted against public databases: NR, GO, eggNOG, Pfam, KEGG, Swiss–Prot, KOG, and COG. Clean reads of each sample were mapped to the *Camellia sinensis* var. sinensis genome. Mapping ratio ranged from 83.85% to 85.75%, respectively, which further indicated that most of the genes were successfully mapped to the tea plant reference genome (Table S3). Further analysis identified 1341 differentially expressed genes (DEGs) in the nine treatment groups (Table S4). As shown in Figure S1, ABA treatment resulted in overall greater transcriptomic changes, with 207, 217, and 142 upregulated and 38, 460, and 610 downregulated genes at 8 h, 72 h, and 168 h after treatment, respectively. To validate the transcriptome result, eight DEGs were selected for qRT–PCR analysis, and these genes had similar expression patterns as those observed in the RNA-Seq results (Figure S2), which indicated that the RNA-Seq results were highly reliable.

#### 3.5.1. GO and KEGG Enrichment Analysis of DEGs

Based on their functions and properties, the DEGs were classified into three major categories: biological process (BP), cellular component (CC), and molecular function (MF) through GO enrichment analysis. Within the BP category, ‘metabolic process’, ‘cellular process’, and ‘single–organism process’ were significantly enriched into the top subcategories (Figure S3). In the CC category ‘catalytic activity’, ‘membrane’, and ‘membrane part’ were found to be more abundant (Figure S3). In the MF category, ‘binding’ and ‘catalytic activities’ were the predominant subcategories. Specifically, comparing DEGs between the ABA treated and water treated, the most GO enrichment occurred in the early period of ABA treatment, which included ‘stimulus’, ‘hormone’, and ‘light stimulus’ responses in the BP category. In the later period of ABA treatment, DEGs involved in ‘regulation of transcription’, ‘anthocyanin-containing compound metabolic process’, and ‘flavonoid metabolic process’ were enriched, followed by those in the MF category including ‘UDP–glycosyltransferase activity’, ‘9–cis–epoxycarotenoid dioxygenase activity’, and ‘transmembrane transporter’.

**Figure 5:**
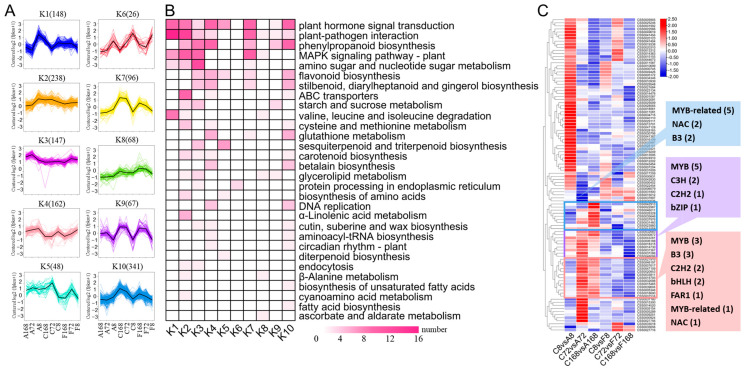
Global expression profile of transcripts in ZXF1 leaves treated with water (C), ABA (A) or Flu (F). k–Means cluster analysis grouped DEGs into 10 clusters (K1–K10) (**A**) where the x axis represents leaf samples collected after 8, 72, and 168 h of each treatment, the y-axis indicates the normalized FPKM, and numbers in parentheses represent DEG numbers within the mentioned cluster. KEGG analysis of 1341 DEGs in the 10 clusters (**B**). The expression pattern of transcription factors after water, ABA or Flu treatments (the number in the parenthesis is the number of genes highly expressed) (**C**).

K-means clustering analysis (Figure 5A) and KEGG enrichment (Figure 5B) were performed to group DEGs with similar expression trends and metabolic pathways into the same clusters. The K–means clustering analysis resulted in 10 distinct clusters, named K1–K10 (Figure 5A; Table S4). Thirty highest DEGs enriched pathways are shown in Figure 5. While inspecting the potential roles of DEGs, the highly expressed genes in ABA treatment group (shown by K1, K2, K3, and K4) were mainly involved in ‘plant hormone signal transduction’, ‘phenylpropanoid biosynthesis’, ‘flavonoid biosynthesis’, ‘ABC transporters’, and ‘carotenoid biosynthesis’. Lowly expressed genes in the Flu treatment group (shown by K5) were mainly involved in ‘plant hormone signal transduction’, ‘flavonoid biosynthesis’, and ‘sesquiterpenoid and triterpenoid biosynthesis’ (Figure 5B; Table S4). Overall, results from GO enrichment, K–means clustering analysis, and KEGG pathway indicated that the differentially expressed genes were mainly involved in phytohormone signaling, phenylpropanoid biosynthesis, flavonoid biosynthesis, ABC transporter, and carotenoid biosynthesis when ZFX1 leaves were treated with either ABA or Flu.

Transcription factors (TFs) are key molecular switches that regulate growth and developmental processes in response to various stresses [33]. A total of 2514 TFs with different expression abundances were detected in ZXF1 leaves, which were classified into 69 families according to the PlantTFDB Database (Figure 5C; Table S5). Among them, WRKY and bHLH families each had 13 members. There were 12 members in MYB and MYB–related families, respectively. AP2/ERF–ERF, C2H2, and NAC each had 8, followed by bZIP (7), MADS–M–type (6), and B3 (5). Some members in WRKY, bHLH, and GRAS families were significantly upregulated after 8 h of ABA treatment. Meanwhile, some TF families were significantly upregulated in 72~168 h after ABA treatment, such as MYB, MYB–related, bHLH, C2H2, B3, and GAR1 (Figure 5C). On the other hand, these TFs were downregulated after Flu treatment, suggesting that these TFs serves a role as regulators from ABA perception to anthocyanin synthesis in ZXF1 leaves.

#### 3.5.2. Exogenous Application of ABA and Flu Affected Anthocyanin Biosynthesis

**Figure 6:**
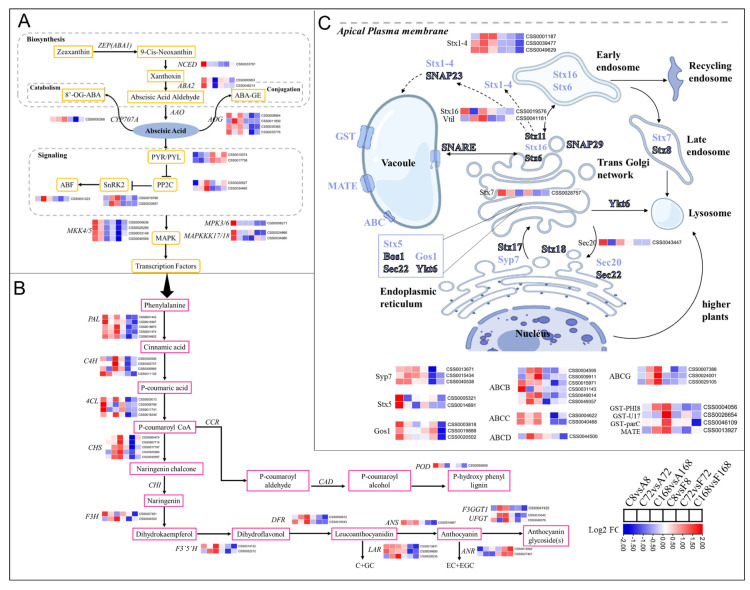
A MapMan overview of DEGs to illustrate ABA, anthocyanin, and vesicle transport after ZXF1 leaves treated with water, ABA or Flu. DEGs are visualized as a heatmap based on log2 fold changes obtained from ABA and Flu treatments against the control treatment. (**A**) DEGs involved in ABA biosynthesis and signal transduction were as follows: NCED, 9–*cis*–epoxycarotenoid dioxygenase; AAO, ascorbate oxidase; CYP707A, ABA 8′–hydroxylase; AOG, abscisate beta–glucosyltransferase; PYR/PYL, pyrabactin resistance–like; PP2C, protein phosphatase 2C; SnRK2, sucrose non–fermenting–1–related protein kinase 2; ABF, ABA-responsive element–binding factors; and MAPK, mitogen–activated protein kinase. (**B**) DEGs involved in flavonoid biosynthesis included PAL, phenylalanine ammonia–lyase; C4H, *trans*–cinnamate 4–monooxygenase; 4CL, 4–coumarate-CoA ligase; CHI, chalcone isomerase; F3H, flavanone 3–hydroxylase, F3′5′H, flavonoid 3′,5′–hydroxylase; LAR, leucocyanidin reductase; DFR, dihydroflavonol 4–reductase; ANS, anthocyanidin synthase; UFGT, UDP–glucose flavonoid 3–O–glucosyltransferase; F3GGT1, Cyanidin 3–O–galactoside 2″–O–xylosyltransferase. (**C**) DEGs from SNARE mediated vesicular trafficking included Bos1, Gos, Qb type golgi SNAP receptor complex; SNAP, soluble NSF attachment protein; SNARE, ‘SNAP REceptor’; Stx, syntaxin–like; Sec20, and vesicle transporter/VAMP–like protein. These genes were differentially expressed in coordination of anthocyanin transport.

To dissect the potential regulation mechanism of exogenous application of ABA or Flu on anthocyanin biosynthesis, we investigated the profiles of DEGs involved in ABA biosynthesis or signal transduction. The key cleavage gene in ABA biosynthesis, *CsNCED* was upregulated in ABA treatments but downregulated in Flu treatment (Figure 6A). *CsCYP707* and *CsAOG* were highly upregulated only under ABA treatment but AOG downregulated in Flu treatment. ABA treatment also induced upregulation of *CsPP2C*, *CsSnRK2*, and *CsABF* as well as carotenoid pathway genes, such as ABA2. ABA2, however, was downregulated in Flu treatment. Additionally, two ABA–receptors pyrabactin resistance–like genes (PYL) were downregulated under ABA treatment (Figure 6A).

To understand the relationship between ABA signal and leaf coloration, transcripts of the DEGs involved in anthocyanin metabolism were compared between ABA and Flu treatments, which are presented via schematic illustration and heatmap insets (Figure 6B). All the major identified unigenes involved in the phenylpropanoid pathway, such as *PAL*, *C4H*, and *4CL* were upregulated upon ABA treatment but downregulated after Flu treatment (Figure 6B). Similarly, all the anthocyanin biosynthetic pathway genes were upregulated in ABA treatment but downregulated in Flu treatment. Two genes, *F3H* and *DFR*, key to the anthocyanin biosynthesis were highly expressed with a log2 fold increase of 1.2 in 8 h and 168 h after ABA treatment, respectively. However, *F3H* had a log2 fold decrease in 3 h after Flu treatment. A key gene involved in the catechin pathway, *ANR*, was highly upregulated in response to Flu but downregulated after ABA treatment. In addition, specific genes for glycosylation modification of anthocyanins, *UFGT*, and *F3GGT* were found to be upregulated after ABA treatment, especially at the later stages of treatment (Figure 6B). After synthesis in the cytoplasm, anthocyanins must be transported to the vacuole for storage and accumulation [34,35]. DEGs identified and annotated as a group of genes related to SNARE–domain family of transporter proteins, such as *Stx5,7,16* (syntaxin), *Sec20,* and *Syp7*, were upregulated in response to the ABA treatment but downregulated after Flu treatment (Figure 6C). The syntaxin genes (*Stx1–4*), usually found in apical plasma membrane, were also upregulated after ABA treatment. Other proteins associated with anthocyanin transport including ABC family of transporter proteins and GSTs transporter were also increasingly expressed by exogenous ABA activation (Figure 6C).

#### 3.5.3. DEGs Involved in Chlorophyll Metabolism and Light Signaling

**Figure 7:**
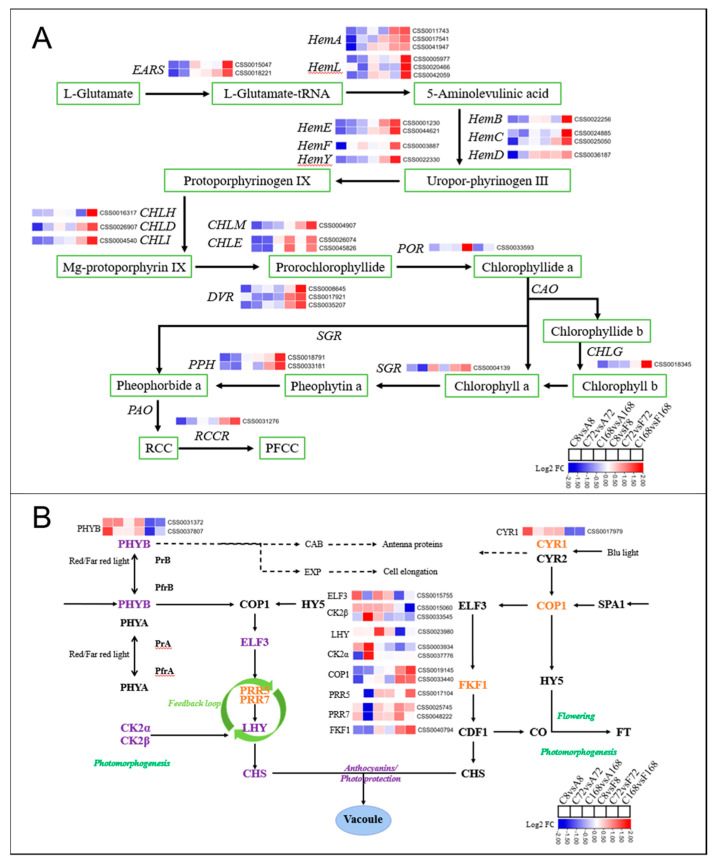
An illustration of DEGs involved in chlorophyll metabolism and light signaling after ZXF1 leaves treated with water, ABA or Flu. DEGs resulting from ABA or Flu treatments versus control treatment are presented in color code boxes based on log2 fold changes. (**A**) The DEGs involved in chlorophyll metabolism included CHLI magnesium chelatase subunit I, CHLM magnesium–prot–porphyrin O–methyltransferase, CAO chlorophyllide an oxygenase; HEMF coproporphyrinogen III oxidase, DVR divinyl chlorophyllide an 8–vinyl–reductase, POR protochlorophyllide reductase, and CHLD chlorophyll/bacteriochlorophyll a. (**B**) The DEGs involved in light signaling included CK2α, casein kinase II subunit alpha; COP1, constitutive photomorphogenic; CRY1/2, cryptochrome; ELF3, early flowering 3; FKF1, flavin-binding kelch domain F box protein; FT, flowering locus T; HY5, elongated hypocotyl 5; PHYA/B, phytochrome; PRR5/7, pseudo response regulator 5/7; and SPA, suppressor of PHYA.

Twenty DEGs were identified as key candidate genes encoding enzymes in chlorophyll metabolism (Figure 7A). Those involved in chlorophyll biosynthesis included: *EARS* (2), *HemA* (3), *HemL* (3), *HemB* (1), *HemC* (2), *HemD* (1), *HemE* (2), *HemF* (1), *HemY* (1), *CHLH* (1), *CHLD* (1), CHLI (1), *CHLM* (1), *CHLE* (2), *DVR* (3), *POR* (1), and *CHLG* (1). Those responsible for chlorophyll degradation were *SGR* (1), *RCCR* (1), and *PPH* (2). These genes were downregulated by ABA application but upregulated by Flu treatment.

Since light played an important role in the biosynthesis of anthocyanins, we analyzed the DEGs of the light signaling pathway. The unigenes corresponding to phytochrome B (*PHYB*) were highly upregulated after ABA treatment, whereas cryptochrome (*CRY1*) transcripts were increasingly expressed after treatment with Flu (Figure 7B). *COP1* expression level was lower after ABA treatment but upregulated with Flu treatment. The expression of *PRR5, PRR7,* and *FKF1* also increased by application of Flu but decreased by ABA application. On the other hand, *CK2α, CK2β,* and *LHY,* key regulators in plant circadian rhythms, were highly upregulated by ABA treatment.

### 3.6. Co-Expressed Gene Networks and Key Candidate Genes Related to Anthocyanin Biosynthesis

**Figure 8:**
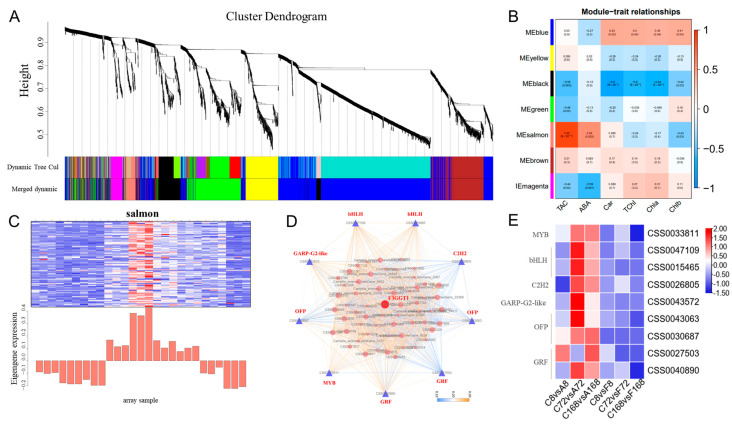
A weighted–gene co–expression network established in ZXF1 leaves after treating with water, ABA or Flu. (**A**) Hierarchical cluster dendrogram constructed by WGCNA, on which each leaf represents a gene. Seven merged modules (based on a threshold of 0.20) identified by weighted-gene co–expression network. (**B**) Module–trait associations based on Pearson correlations. Each row represents one module, and the gene numbers in each module are shown on the far left. Each column corresponds to one metabolite. (**C**) Expression pattern of genes in MEsalmon. (**D**) Gene co-expression network for the MEsalmon module. All of the 85 genes in this module were used to construct the co–expression network and were visualized by Cytoscape 3.8.2 software. The purple triangles and red circle represent the key hub genes. (**E**) Heatmap of salmon module TF family in different treatment groups. The TF family from ABA and Flu treatment versus control contrasts are presented in color code boxes based on log2 fold changes.

A weighted gene co-expression network analysis (WGCNA) was used to build a co-expression network and identify the hub genes regulating anthocyanin synthesis. A total of seven co–expression modules (labeled and highlighted using different colors) were identified (Table S6). The MEsalmon gene set was significantly correlated with ABA accumulation and changes in anthocyanin (Figure 8; Table S6). The genes clustered in the MEsalmon module showed relatively higher expression in the ABA treatment group. The correlation analysis showed that the gene significance and module membership in salmon modules had the highest correlation coefficients of 0.9 (*p* = 1.4 × 10^−34^) (Figure 8B). KEGG analysis of genes in ‘salmon’ modules revealed that all significantly enriched metabolism pathways were related to amino sugar and nucleotide sugar metabolism, phenylpropanoid biosynthesis, flavonoid biosynthesis, flavone and flavonol biosynthesis (Figure S4; Table S6). The result suggested that genes in this module participated in the synthesis and metabolism of compounds related to anthocyanins.

To select the hub genes related to anthocyanin synthesis, we investigated the TFs and anthocyanin–related genes in ‘salmon’ modules. Based on the kME values, the top 150 genes in the ‘salmon’ module were used to construct the co–expression network, and among them, the TFs were selected as key hub genes. As shown in Figure 8D, nine TFs and one anthocyanin-related genes were identified in the salmon module, including *MYB* (CSS0033811), *bHLH* (CSS0047109 and CSS0015465), *C2H2* (CSS0026805), *GARP*–*G2*–*like* (CSS0043572), *OFP* (CSS0043063 and CSS0030687), *GRF* (CSS0027503 and CSS0040890), and *F3GGT1* (CSS0047425). Notably, all these genes were highly expressed after ABA treatment, especially from 72 h to 168 h (Figure 8E). These results indicated that the ABA signal could directly induce the expressions of responsive factors, leading to the upregulation of anthocyanin–related genes in ZXF1 leaves.

## 4. Discussion

This study was conducted in a tea plantation in Wuyi Mountain, one of the most important tea production regions in China. Our survey showed that the occurrence in purplish leaves in ZFX1 is due largely to the response of plants to high light as September had the highest light intensity although the temperature was the highest in this month as well. High radiation–induced color changes in tea leaves were also reported [36], suggesting that the leaf color of purple tea is not set in stone. High light intensity can induce plant oxidative. Studies have shown that the scavenging of reactive oxygen species (ROS), especially hydrogen peroxide (H_2_O_2_), depends mainly on non–enzymatic antioxidants [37]. Anthocyanin molecules in the vacuoles are implicated in H_2_O_2_ scavenging during stress [38]. Analyses of the leaf extracts from our shade experiment showed that high antioxidant activities were significantly correlated with anthocyanin contents. The onset synthesis and accumulation of anthocyanin may allow plants to restore the redox balance and reduce the risk of oxidative stress [39].

Subsequent analyses indicated that leaf purpleness and contents of anthocyanin and ABA are positively correlated (Figure 1 and Figure 3). The high correlation (Figure 1) may suggest that high light–triggered ROS activate both anthocyanin and ABA biosynthesis. ROS have been shown to act as second messengers for hormone signaling including ABA [40]. On the other hand, ABA also enhanced anthocyanin synthesis and accumulation (Figure 2 and Figure 3). This was probably attributed to ABA application’s resultant production of H_2_O_2_ that triggered anthocyanin biosynthesis. Several studies with *Arabidopsis* have demonstrated that application of ABA can induce generation of H_2_O_2_ [41,42]. H_2_O_2_ then enhances anthocyanin accumulation via upregulation of anthocyanin related genes [43]. Thus, anthocyanins act as antioxidant molecules alleviating oxidative stress of plants. Additionally, we found that ABA treatment also resulted in a significant decrease in chlorophyll and carotenoids in ZFX1 leaves. These results suggest that the increase in anthocyanin content interferes chlorophyll synthesis, while carotenoid accumulation was also inhibited. On the other hand, ABA treatment led to the catabolic conversion of carotenoids, which also contributed to the decrease in carotenoids content [44]. The production of ABA in plants was inhibited by treatment with Flu, which blocks ABA-biosynthesis at the step of phytoene desaturase (*PDS*) [45]. In the present study, a significant decrease in the content of ABA was noticed at 72 h after Flu treatment. At the same time, the content of anthocyanins was rapidly decreased (Figure 4), which is consistent with previous results [22]. In addition, the decrease in carotenoids and chlorophyll contents at 8 h after Flu treatment (Figure 4) suggests that the inhibition of carotenoid synthesis by Flu precedes ABA. It is likely that when leaf anthocyanin content is reduced, there is not enough carotenoids in the leaves as supplementary substances for photoprotection. Therefore, chlorophyll is degraded by photooxidation, resulting in the tea leaves with reduced greenness [46,47].

To elucidate molecular mechanisms behind anthocyanin accumulation in young leaves of ZFX1, plants treated with ABA or Flu, along with water as the control, were used for identifying DEGs through RNA-Seq. The ABA treatment upregulated all the anthocyanin biosynthetic genes in young leaves, including the bottleneck flavonoid biosynthetic genes CHS, ANS, and 3GT described in tea plants [48,49,50]. However, the results from Flu treatment were the opposite. As a result, there was a higher accumulation of anthocyanins after ABA treatment compared to the control treatment. Furthermore, the expression levels of *UFGT, F3GGT1, F3H,* and *DFR* highly increased after ABA application. This is consistent with the findings of previous studies [22]. In bright–colored fruits, genes encoding key enzymes downstream of the anthocyanin biosynthesis pathway, such as *DFR, ANS,* and *UFGT*, are often highly expressed [51]. It is known that the expression of *UFGT* genes is regarded as one of the most important regulated steps in the anthocyanin biosynthetic pathway [52,53]. In anthocyanin biosynthesis pathway, the first stable product is formed by the action of glycosyltransferase to catalyze the transfer of a sugar molecule to the hydroxyl group in position 3 of anthocyanidin aglycone. It has been reported that anthocyanin concentration and flavonoid–3–O–glucosyltransferase activity increase parallelly during fruit ripening [54]. Additionally, the transportation of anthocyanins into vacuoles is usually trafficked intra–cellularly by different classes of transporter proteins, such as MATEs, ABCs, and GSTs [55]. However, responses of transporters in delivery of anthocyanins in tea plants have not been studied. In the present study, RNA–Seq demonstrated that a set of transporter genes, including *Stx, Gos1, Sec20, Syp7*, ABC transporters, MATE, and GSTs were highly upregulated in response to ABA treatment but downregulated in response to Flu treatment. Our results also show the potential role of syntaxin genes in response to ABA-induced anthocyanin transport mechanisms. These different types of SNAREs, barring the ER-localized Syp and Sec, are usually localized in endosomes and in trans–Golgi network [56]. The proposed vesicular trafficking model for anthocyanins in tea plants might also be coregulated by other GSTs, MATE efflux transporters, and ABC transporters.

Based on the transcriptome data, all the chlorophyll metabolic genes in tea leaf were downregulated after ABA treatment, including the bottleneck chlorophyll metabolic genes *HemA, HemL, CHLH, PAO,* and *RCCR* described in tea plants [57,58]. Our results are in agreement with the study in grapes [59]. During leaf senescence, exogenously applied ABA can induce senescence associated mRNAs that cause degreening or yellowing of leaves due to chlorophyll degradation [60,61]. However, this result differs from previous reports on litchi, which concluded that ABF recognized ABA–responsive elements in the promoter region of Chl degradation–related genes (*PAO* and *SGR*) and promoted chlorophyll degradation [62]. An explanation for this observation might be that tea plants had different regulatory mechanisms for ABA. Bagging experiments have revealed that sunlight is essential for anthocyanin synthesis in tea plants [11,63]. Sunlight affects both anthocyanin accumulation and the expression of both anthocyanin biosynthetic genes and/or regulatory genes in tea plants. It is well known that the green leaves process the excess radiant energy effectively, and the purple leaves optimize light harvesting and photoprotection. Thus far, anthocyanins can work as light attenuators or antioxidants plays a protective role against photooxidative stress [43]. H_2_O_2_ is a key second messenger in the light–ABA signal response to promote stomatal closure. Tattini compared the physiological response and the expression of genes involved in light signaling between green (‘*Tigullio*’, TIG) and red (‘Red Rubin’, RR) basil. ABA glucose ester deglycosylation and reduced ABA oxidation caused the photosynthesis decrease in green TIG because of larger stomatal limitations [64].

In plants, anthocyanin synthesis-related genes are primarily regulated at the transcriptional level [65]. Based on the research on pigment and related metabolic pathway genes in tea plants, the biosynthesis of anthocyanins occurred throughout the entire process of ABA treatment, and the genes encoding enzymes associated with anthocyanin biosynthesis are transcriptionally regulated by transcription factors. In the WGCNA network, *CsF3GGT1* was highly related with *CsMYB3*. *F3GGT* is a class of glycosyltransferases involved in the final step of anthocyanin synthesis and in kiwifruit, *F3GGT* was shown to be highly correlated with anthocyanin synthesis [66]. Previous studies have shown that the MYB–bHLH–WDR (MBW) regulatory complex coordinately activates multiple genes related to anthocyanins [67,68]. Some of the members of MYB have been identified to be the major determinant regulators in the MBW complex involved in anthocyanin biosynthesis. The *CsMYB3* homolog *AtMYB113* (97% identity in amino acid sequence, Figure S6A) in *Arabidopsis* activated the expressions of almost all flavonoid pathway genes, resulting in an increase in both flavonol and anthocyanin contents [69]. Another study indicated that ABA affected the upstream gene *LcMYB1*, which regulates *LcUFGT* expression and subsequently the accumulation of anthocyanins in the pericarp of litchis [70]. In sweet cherries, it was shown that *PavMYB10.1* positively regulate anthocyanin accumulation by interacting with *PavbHLH* and *PavWD40* and binding to the promoter regions of *PavANS* and *PavUFGT* [71]. In purple tea, the action of *CsMYB75* results in the selective upregulation of anthocyanins and this selective regulation was achieved through *CsGSTF1* [49]. The upregulation of *CsMYB3* after ABA treatment and its similar expression with *CsF3GGT1* also support this conclusion. It is documented that the myb–homologous C1 require a member of the bHLH–containing R or B gene family to activate transcription of the anthocyanin biosynthetic genes [72]. Here, two bHLH genes (CSS0047109 and CSS0015465) showed a high correlation with *CsMYB3* and *CsF3GGT1*, which are homologous to *AtbHLH94* (100% identity in amino acid sequence, Figure S6B) and regulated by the ABA signaling pathway [73]. In litchis, transcription levels of LcbHLHs were inconsistent with anthocyanin accumulation in different tissues and during fruit development, while both *LcbHLH1* and *LcbHLH3* enhanced anthocyanin accumulation through coinfiltration with *LcMYB1* [74]. Our results showed that bHLHs might interact with MYB for regulating anthocyanin accumulation. In addition, *bHLH6* (*MYC2*) TF is reported to be commonly involved in light and ABA signaling pathways [75]. We believed that *CsMYB3* and two bHLH genes (CSS0047109 and CSS0015465), which were highly induced at 72~168 h after ABA treatment, may be involved in metabolic reactions following the reception of ABA signal.

## 5. Conclusions

**Figure 9:**
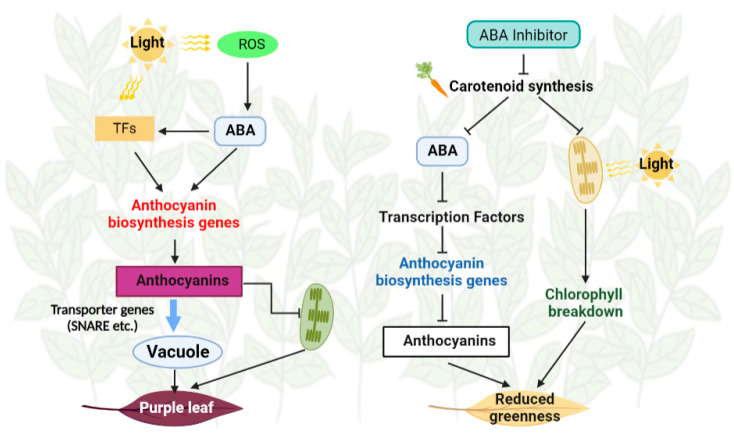
A proposed model on the role of ABA in regulating color changes in young leaves of ZXF1 plants. Exogenous ABA induces the expression of CsMYB4/44 and further induces bHLH and MYB expression. Subsequently, these TFs directly activate the expression of anthocyanins biosynthesis and transport genes. Chlorophyll synthesis was inhibited, resulting in the tea leaves turning purple. Additive effects were observed in anthocyanin accumulation under combined light and ABA. On the other hand, exogenous Flu suppressed ABA synthesis and reduced the expression of genes in anthocyanin biosynthesis. At the same time, chlorophyll was degraded by photooxidation, resulting in the tea leaves with reduced greenness. The arrows and blunt–head lines indicate activation and suppression, respectively.

The present study shows that anthocyanin accumulation in young leaves of ZFX1 is mainly attributed to plant responses to high light intensity. Anthocyanins are antioxidant molecules that alleviate oxidative stress and scavenging free radicals and ROS. Increased anthocyanin is also parallelized with elevated levels of ABA. Based on the above analysis, a model for anthocyanin accumulation in ZFX1 cultivar was proposed (Figure 9). High light intensity may cause reactive oxygen stress, which triggers the biosynthesis of ABA. ABA promoted the expression of transcription factors, such as MYB, which in turn promoted the expression of anthocyanin biosynthesis genes, enhanced anthocyanin biosynthesis, limited chlorophyll and carotenoid accumulation. Meanwhile, genes encoding transporter proteins were highly upregulated, resulting in the transport of anthocyanin into vacuoles and young leaves of ZFX1 displayed as purplish coloration, whereas leaves treated with Flu had opposite action. Our further research with ZFX1 is warranted to validate this model.

## Data Availability

The data that support the findings of this study are openly available in the National Center for Biotechnology Information (NCBI) SRA database under the BioProject ID: PRJNA910856.

## Supplementary Materials

The following supporting information can be downloaded at: https://www.mdpi.com/article/10.3390/antiox12020392/s1, Figure S1: DEGs in ZFX1 leaves in response to ABA and Flu treatment. (A) Correlation analysis among three replicates of the transcriptome data. (B) 2D scatter plot of three treatment groups in which individual expression differences were tested via principal component analysis (PCA). The three oval areas show the different subgroups: red represents the control, green is ABA treatment, and blue corresponds to flu treatment. (C) Functional annotations of genes in the indicated databases. (D) The number of DEGs between ABA treatment and Flu treatment in the indicated groups; Figure S2: qRT–PCR analysis of key genes and validation of DEGs by Pearson’s linear correlation. (A) qRT–PCR analysis of tea carotenoid-ABA pathway genes (*CsNCED, CsPP2C*), flavonoid biosynthetic genes (*CsPAL, CsUFGT*), vesicular trafficking genes (*CsStx1–4, CsABCB1*), chlorophyll metabolism gene (*CsRCCR*), and transcription factor (*CsMYB*). Error bars represent standard error (±SE) of minimum three biological replicates. (B) Pearson’s linear correlation plot by comparison between select key gene expression results from qPCR and DEGs from RNA–seq dataset, which are expressed in log2fold changes. FPKM: Fragments Per Kilobase of exon model per Million mapped fragments; Figure S3: GO enrichment analysis of DEGs between six comparison subsets. The genes were categorized based on gene ontology annotation, and the proportion of each category was displayed in the categories of biological process (BP), cellular component (CC), and molecular function (MF); Figure S4: GO–enrichment analysis of module salmon; Figure S5: Scatterplot of gene significance score versus module membership in the blue module (A), black module (B), magenta module (C), yellow module (D), salmon module (E), green module (F), and brown module (G); Figure S6: Expression heatmaps and the consensus profiles of co–expressed genes in different modules; Figure S7: Identification of known MYB transcription factors regulating anthocyanin synthesis in tea plants; Table S1: Primers used in this study; Table S2: Sequencing data Statistics; Table S3: Statistics on data mapping; Table S4: DEGs of tea plant (*Camellia sinensis* (L.) O. Kuntze) among nine treatment group; Table S5: Expression of TFs at nine treatment group; Table S6: Data from WGCNA analysis.
