# Supplementary material for: High Light Intensity Triggered Abscisic Acid Biosynthesis Mediates Anthocyanin Accumulation in Young Leaves of Tea Plant (Camellia sinensis)
Source: Antioxidants (Basel). 2023 Feb 6;12(2):392. doi: 10.3390/antiox12020392 (PMC9952078; doi:10.3390/antiox12020392)
Supplement: Supplementary file 1 [file antioxidants-12-00392-s001.zip › antioxidants-2162831-supplementary-final/antioxidants-2162831-supplementary-highlight done.pdf]

Supporting Information

High Light Intensity Triggered Abscisic Acid Biosynthesis Mediates Anthocyanin Accumulation in Young Leaves of Tea Plant (*Camellia sinensis*)

Chenxi Gao[1], Yue Sun[1], Jing Li[1], Zhe Zhou[1], Xuming Deng[1], Zhihui Wang[1], Shaoling Wu[1], Lin Lin[2], Yan Huang[2], Wen Zeng[1], Shiheng Lyu[1], Jianjun Chen[3], Shixian Cao[4], Shuntian Yu[4], Zhidan Chen[2,*], Weijiang Sun[1,*], Zhihui Xue[2,*]

1 College of Horticulture, Fujian Agriculture and Forestry University, Fuzhou, 350002, China
2 Anxi College of Tea Science, Fujian Agriculture and Forestry University, Quanzhou, 362400, China
3 Mid-Florida Research and Education Center, Department of Environmental Horticulture, Institute of Food and Agricultural Sciences, University of Florida, Apopka, FL 32703, USA
4 Wuyixing Tea Industry Co., Ltd., Nanping, 353000, China
* Correspondence: chenzhidan@fafu.edu.cn (Z.C.); sunweijiang@hotmail.com (W.S.); xzh@fafu.edu.cn (Z.X.); Tel.: +86-15805998677 (Z.C.); +86-13705067139 (W.S.); +86-13405916632 (Z.X.)

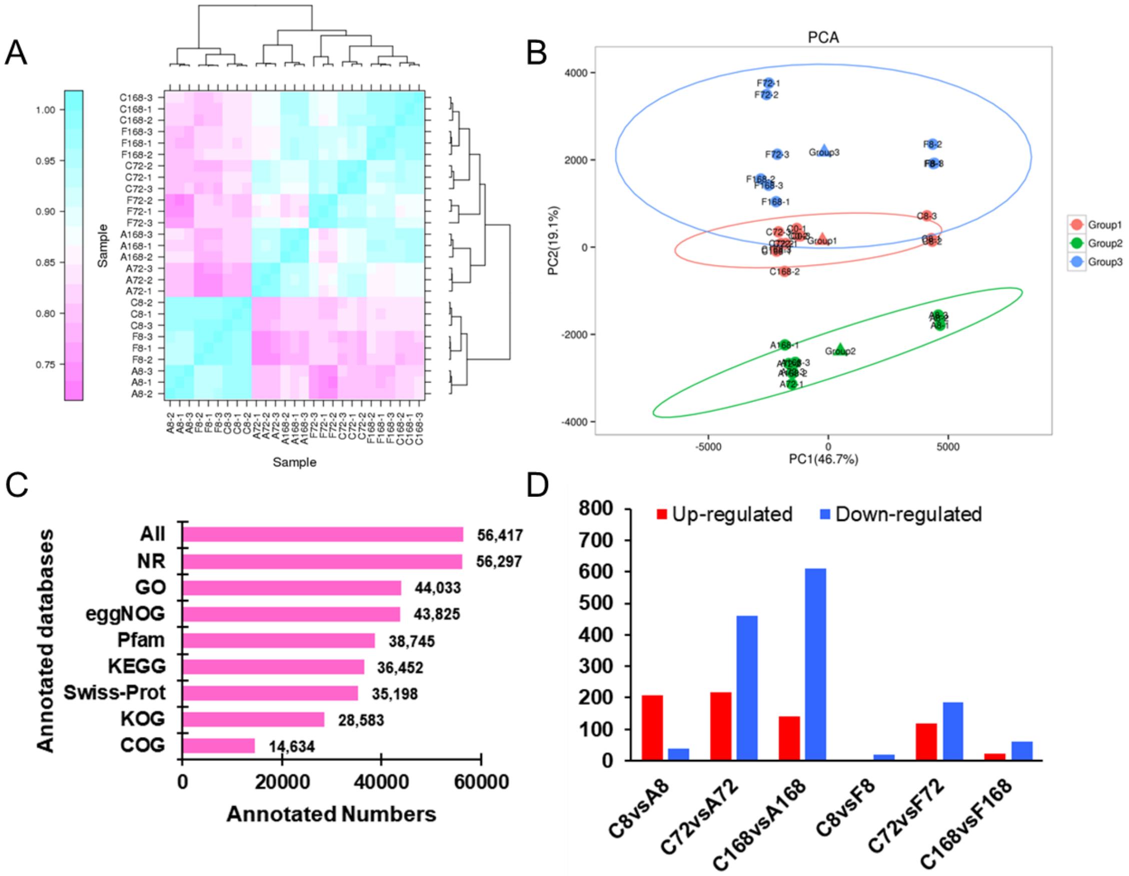

Figure S1. DEGs in ZFX1 leaves in response to water (C), ABA (A) or Flu (F) treatment after 8, 72, and 168 h of treatment. (A) Correlation analysis among three replicates of the transcriptome data across treatments. (B) 2D scatter plot of three treatment groups in which individual expression differences were tested via principal component analysis (PCA). The three oval areas show the different subgroups: red represents the control (water treatment), green is ABA treatment, and blue corresponds to flu treatment. (C) Functional annotations of genes in the indicated databases. (D) The number of upregulated and downregulated DEGs resulted from the comparison between ABA or Flu with the water treatment at the indicated sampling time.

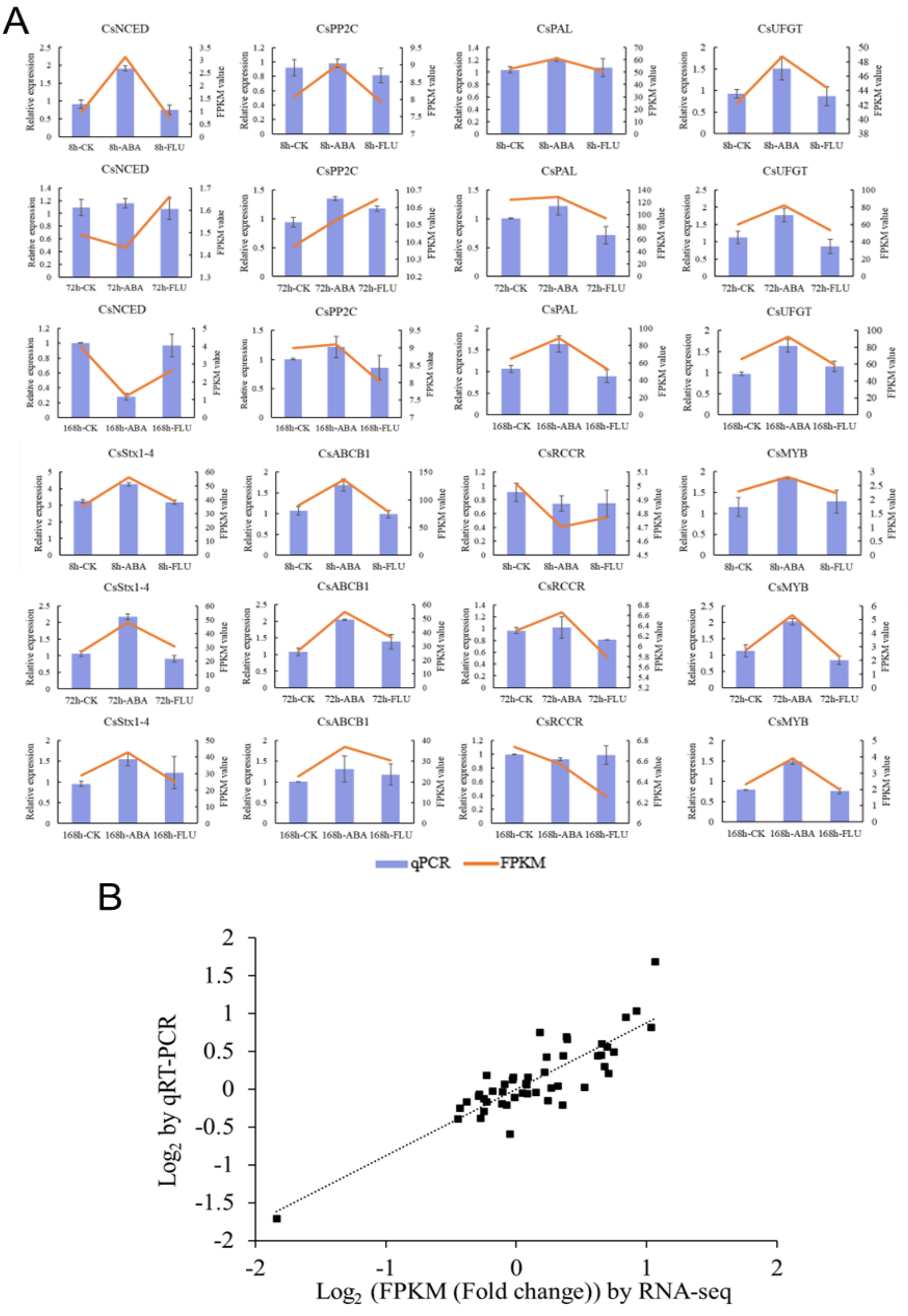

Figure S2. qRT-PCR analysis of key genes and validation of DEGs by Pearson's linear correlation. (A) qRT-PCR analysis of tea carotenoid-ABA pathway genes (*CsNCED, CsPP2C*), flavonoid biosynthetic genes (*CsPAL, CsUFGT*), vesicular trafficking genes (*CsStx1-4, CsABCB1*), chlorophyll metabolism gene (*CsRCCR*), and transcription factor (*CsMYB*). Error bars represent standard error (± SE) of three biological replicates. (B) Pearson's linear correlation plot by comparison the expression of select key genes analyzed by qPCR with DEGs from RNA-seq dataset. The expression is based on log2fold changes. FPKM (Fragments Per Kilobase of exon model per Million mapped fragments).

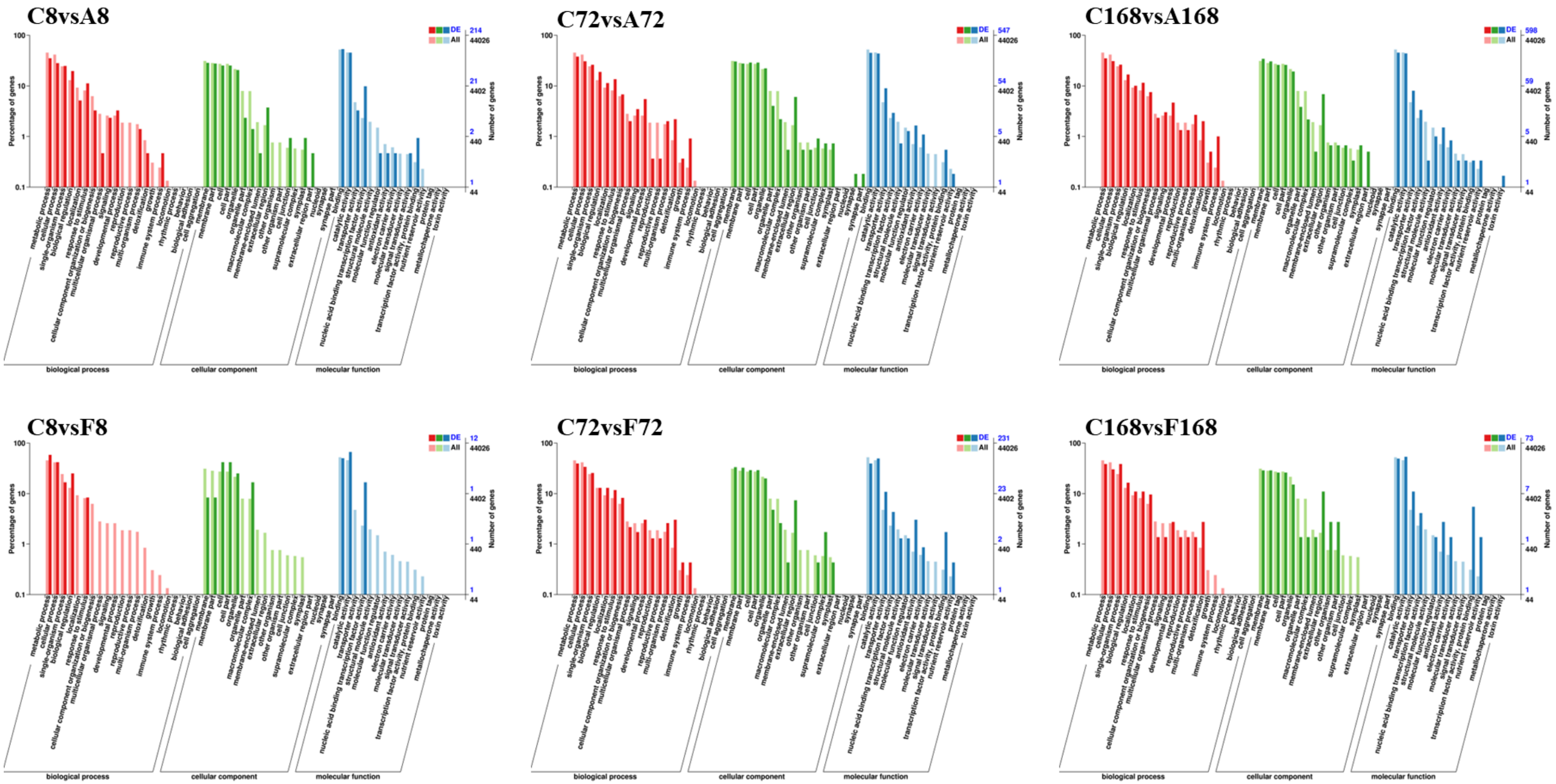

Figure S3. GO enrichment analysis of DEGs between six comparison subsets. The genes were categorized based on gene ontology annotation, and the proportion of each category was displayed in the categories of biological process (BP), cellular component (CC), and molecular function (MF) where ZFX1 leaf samples were collected after 8, 72, and 168 h of water (C), ABA (A), and Flu (F) treatments, respectively.

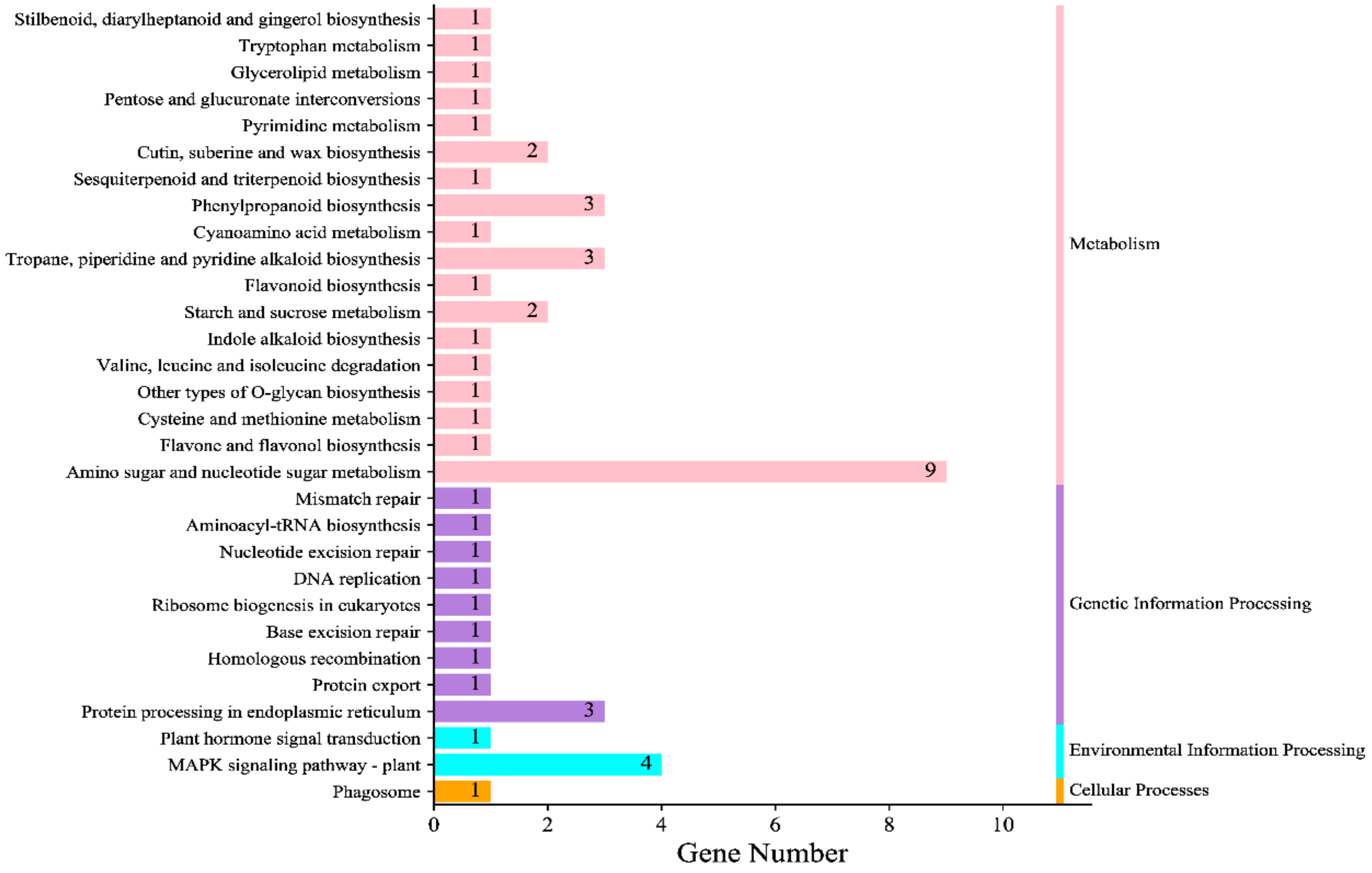

Figure S4. GO-enrichment analysis of module salmon.

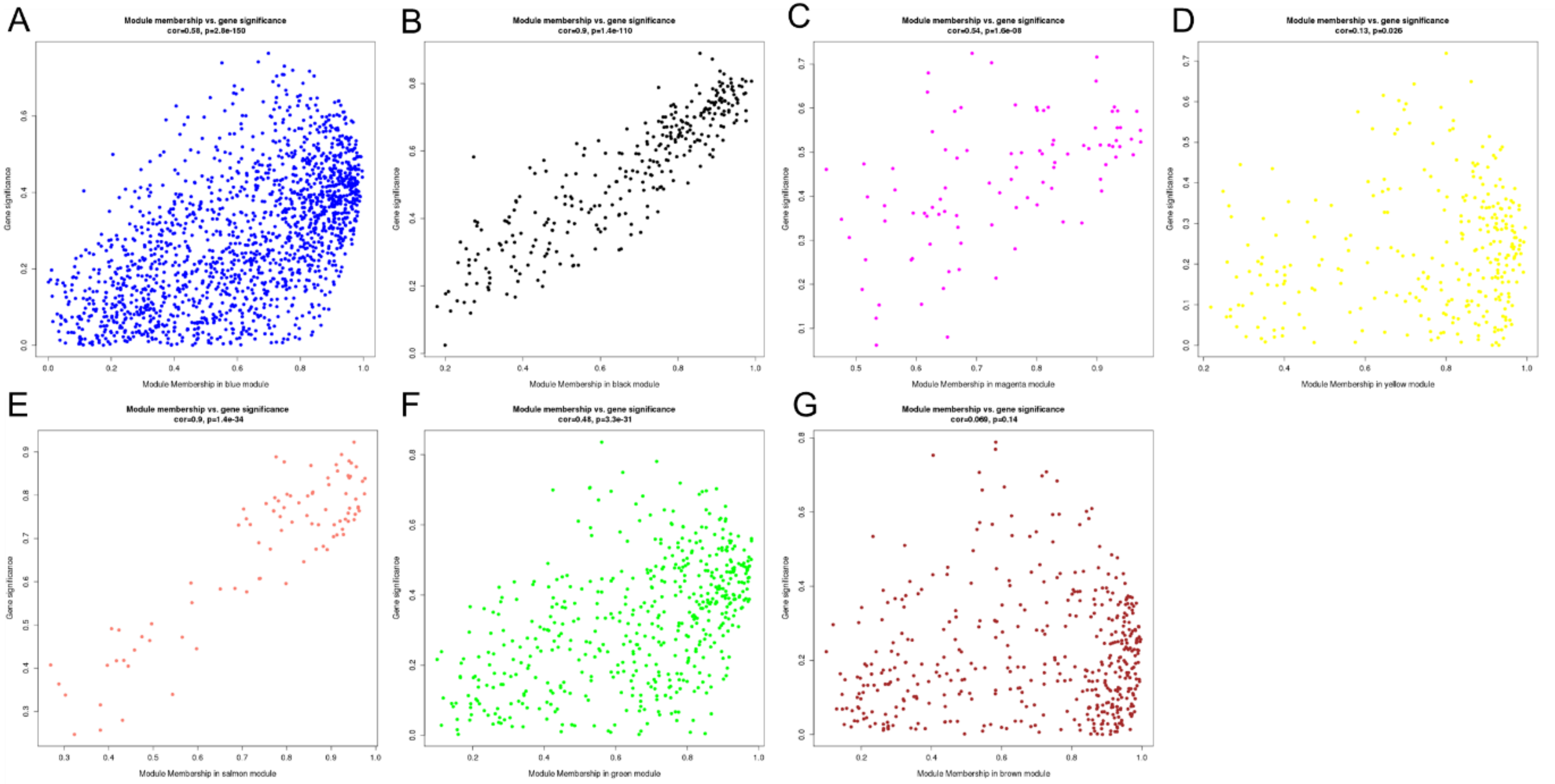

Figure S5. Scatterplot of gene significance score versus module membership in the blue module (A), black module (B), magenta module (C), yellow module (D), salmon module (E), green module (F), and brown module (G).

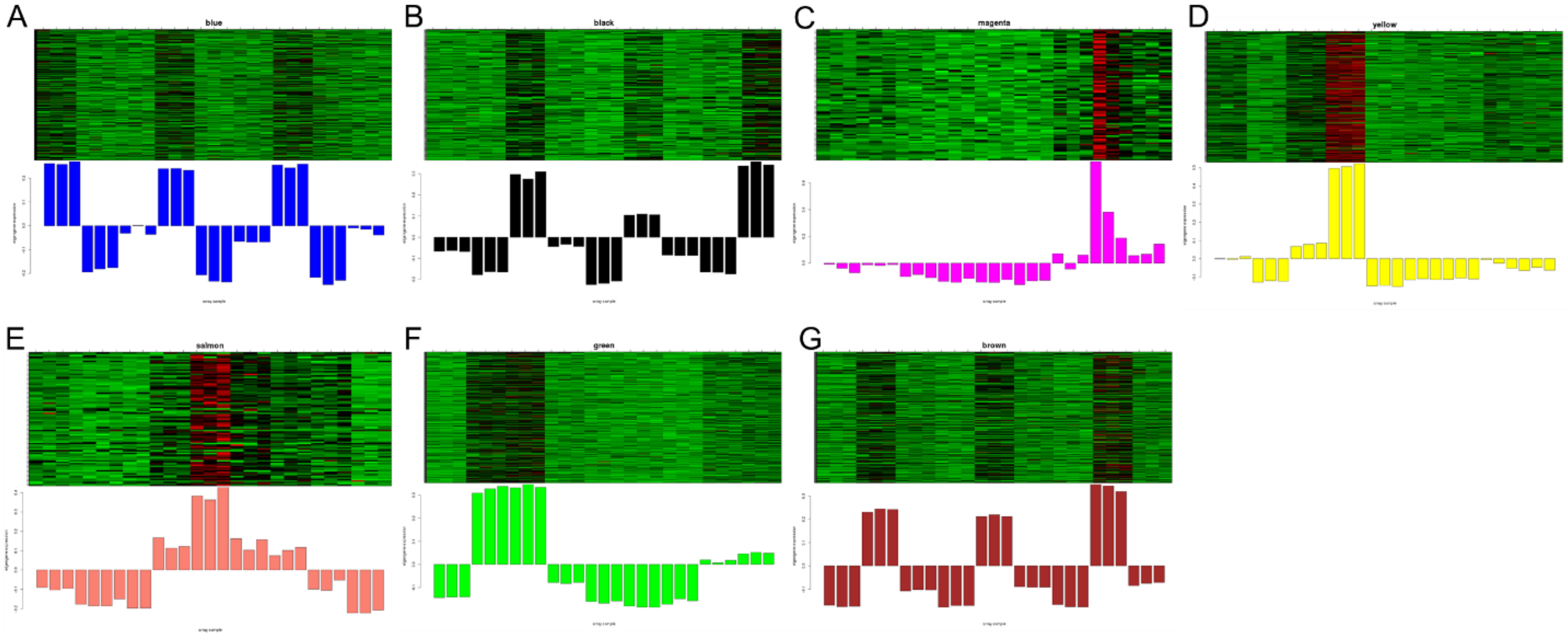

Figure S6. Expression heatmaps and the consensus profiles of co-expressed genes in different modules.

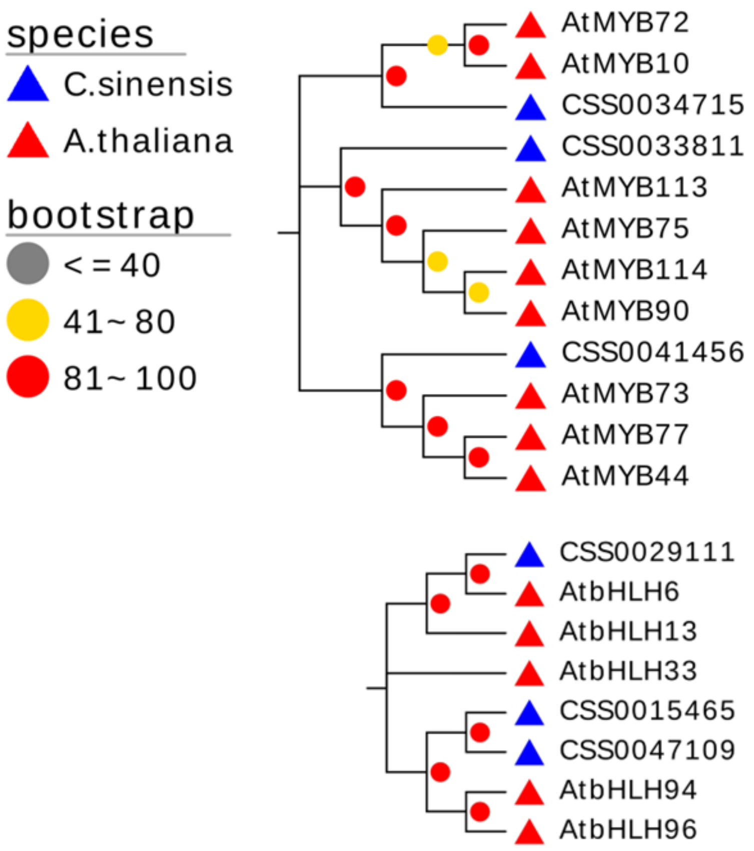

Figure S7. Identification of known MYB transcription factors regulating anthocyanin synthesis in tea plants.
